# Improved accuracy assessment for 3D genome reconstructions

**DOI:** 10.1186/s12859-018-2214-2

**Published:** 2018-05-30

**Authors:** Mark R. Segal, Henrik L. Bengtsson

**Affiliations:** Division of Bioinformatics, Department of Epidemiology and Biostatistics, UCSF, 16th Street, San Francisco, 94158 USA

**Keywords:** Chromatin conformation capture, Multiplex FISH, Genome architecture mapping, Procrustes alignment, Principal components analysis

## Abstract

**Background:**

Three dimensional (3D) genome spatial organization is critical for numerous cellular functions, including transcription, while certain conformation-driven structural alterations are frequently oncogenic. Genome conformation had been difficult to elucidate but the advent chromatin conformation capture assays, notably Hi-C, has transformed understanding of chromatin architecture and yielded numerous biological insights. Although most of these findings have flowed from analysis of proximity data produced by these assays, added value in generating 3D reconstructions has been demonstrated, deriving, in part, from superposing genomic features on the reconstruction. However, advantages of 3D structure-based analyses are clearly conditional on the accuracy of the attendant reconstructions, which is difficult to assess. Proponents of competing reconstruction algorithms have evaluated their accuracy by recourse to simulation of toy structures and/or limited fluorescence in situ hybridization (FISH) imaging that features a handful of low resolution probes. Accordingly, new methods of reconstruction accuracy assessment are needed.

**Results:**

Here we utilize two recently devised assays to develop methodology for assessing 3D reconstruction accuracy. Multiplex FISH increases the number of probes by an order of magnitude and hence the number of inter-probe distances by two orders, providing sufficient information for structure-level evaluation via mean-squared deviations (MSD). Crucially, underscoring multiplex FISH applications are large numbers of coordinate-system aligned replicates that provide the basis for a referent distribution for MSD statistics. Using this system we show that reconstructions based on Hi-C data for IMR90 cells are accurate for some chromosomes but not others. The second new assay, genome architecture mapping, utilizes large numbers of thin cryosections to obtain a measure of proximity. We exploit the planarity of the cryosections – not used in inferring proximity – to obtain measures of reconstruction accuracy, with referents provided via resampling. Application to mouse embryonic stem cells shows reconstruction accuracies that vary by chromosome.

**Conclusions:**

We have developed methods for assessing the accuracy of 3D genome reconstructions that exploit features of recently advanced multiplex FISH and genome architecture mapping assays. These approaches can help overcome the absence of gold standards for making such assessments which are important in view of the considerable uncertainties surrounding 3D genome reconstruction.

## Background

Genome conformation is critical for numerous cellular processes, including gene regulation, while certain conformation-driven structural alterations (e.g. translocations, fusions) are frequently oncogenic. Until recently, genome conformation had been notoriously difficult to interrogate. However, the emergence of the suite of chromatin conformation capture assays, notably Hi-C, has transformed understanding of chromatin architecture and yielded numerous downstream biological insights [2, 8, 9, 17, 24]. The data resulting from these assays, typically performed on large cell populations, are generally depicted as *contact* or *interaction* (heat)maps, which record the frequency with which pairs of genomic loci are cross-linked, reflecting spatial proximity of the respective loci within the nucleus. Many novel conformational-related findings have flowed from direct analysis of such contact level data. But, by converting contact frequencies into distances (typically assuming inverse power-law relationships [2, 13, 27, 29]), it is possible to generate a three dimensional (3D) reconstruction of the associated chromatin architecture via versions of the multi-dimensional scaling (MDS) paradigm. There have been several demonstrations of the added value of performing downstream analysis based on a 3D representation rather than the attendant contact map, these benefits deriving from the ability to superpose genomic features on the reconstruction. Examples include co-localization of genomic landmarks such as early replication origins in yeast [4, 32], gene expression gradients in relation to telomeric distance and co-localization of virulence genes in the malaria parasite *Plasmodium falciparum* [2], the impact of spatial organization on double strand break repair [15], and elucidation of ‘3D hotspots’ corresponding to (say) overlaid ChIP-Seq transcription factor extremes which can reveal novel regulatory interactions [5].

Yet, such putative advantages of 3D structure-based analyses are clearly conditional on the accuracy of the corresponding reconstruction and there are many reasons why such accuracy can and has been questioned. First, the very notion of a single genome architecture being representative of the large (∼10^6^) cell populations on which Hi-C assays are performed is highly simplified [20]. This concern has spawned several reconstruction approaches [13, 28] wherein an ensemble of solutions is generated, intended to reflect inter-cell variation. However, as has been noted [26, 29], whether these collections capture biologic variation is unclear, since reconstruction differences could equally be algorithmic. The recent development of high-throughput *single-cell* Hi-C assays [23], utilizing multiplexing via combinatorial cellular indexing [6], affords the possibility of systematically studying this issue. Here, we will assume that a consensus reconstruction provides a meaningful summary, but address associated reproducibility across replicate data series. Second, there are a multitude of competing reconstruction algorithms, each with a number of tuning parameters and little theoretic basis for arbitrating between them. The absence of gold standards makes empiric comparisons problematic: while some authors have appealed to simulation [16, 22, 29, 33, 34], real data referents remain desirable. To that end, many of the same reconstruction algorithm developers have made recourse to fluorescence in situ hybridization (FISH) imaging as a means for gauging the accuracy of competing algorithms and/or tuning parameter settings. This proceeds by comparing distances between imaged probes with corresponding reconstruction-based distances. However, such methods are tenuous at best due to the limited number of probes (∼2−6, [17, 22, 27]) and the modest resolution thereof, many straddling over 1 megabase (Mb).

Here we show how newly devised biotechnologies can dramatically improve 3D genome reconstruction accuracy assessment and demonstrate computational and statistical techniques for realizing this promise. The advent of multiplex FISH [30] has the potential to advance 3D genome reconstruction accuracy evaluation by furnishing detailed gold standards. This derives from multiplex FISH providing an order of magnitude more probes, each at higher resolution, and hence two orders of magnitude more distances than conventional FISH. All Hi-C related techniques rely on proximity-based ligation whereas ligation-free methods, notably genome architecture mapping (GAM), confer several advantages [3]. GAM is predicated on sequencing DNA from a large collection of randomly-oriented, thin nuclear cryosections then determining co-segregation which, in turn, yields a contact matrix analog. By using such matrices to generate a 3D genome reconstruction we can obtain an internal measure of accuracy by assessing how well the reconstruction conforms to the underlying collection of planar nuclear cryosections as described in Methods.

## Methods

### Data Acquisition and Pre-processing

We obtained multiplex FISH probe coordinates for chromosomes 20, 21, and 22 for IMR90 cells from Figures & Data at https://doi.org/10.1126/science.aaf8084. The numbers of probes, centered at previously defined topologically associated domains (TADs, [8]) was respectively 30, 34, and 27. Importantly, as we will subsequently exploit, numerous multiplex FISH replicates for each chromosome were available, respectively 111, 120, and 151. However, variable patterns of low-level (<5*%*) probe missingness pertained over the replicates. We handled this via imputation – simply averaging over non-missing (aligned) coordinates of the corresponding probes across replicate chromosomes – and discuss implications of this approach under Accuracy Assessment below. Crucial to this imputation approach was the fact that the chromosome replicates were imaged using a common coordinate system [30].

While multiplex FISH probe coordinates are also available for active and inactive X chromosomes we are not positioned to evaluate corresponding 3D reconstruction accuracy since the associated Hi-C data does not differentiate between active and inactive X. Given the multiplex FISH data, deconvolving the Hi-C X chromosome data into active and inactive counts would be possible, but then we couldn’t deploy the same multiplex FISH data for accuracy assessment.

Hi-C data [8] for IMR90 cells was obtained from the Gene Expression Omnibus (GEO) with accession GSE35156. Contact matrices deriving from several series of experiments were grouped (by the original authors) into ‘primary’ and ‘replicate’ datasets and we utilize these both separately and together as described below in Obtaining 3D Genome Reconstructions. Based on data at 5 kilobase (kb) resolution we coarsened, via binning, to obtain data at 25kb, 50kb and 100kb resolution, finer resolutions not being purposeful in view of the referent multiplex FISH resolution.

GAM data for mouse embryonic stem cells (mESC) was obtained from GEO with accession GSE64881. Available data include measures of proximity (‘normalized linkage disequilibrium’ scores) which, as demonstrated [3], are highly correlated with Hi-C contacts, despite the former being obtained using ligation-free methods. Accordingly, we treat these scores analogously to contact matrices and use them as inputs to a 3D genome reconstruction algorithm. However, the particular algorithm we focus on, HSA (see next), requires that input scores be non-negative, which we achieve here by simple translation such that the resultant minimum score is zero. Also available, and crucial for our approach to accuracy assessment, is the binary segmentation matrix identifying which genomic region (locus; row) was observed in which nuclear profile (cryosection; column). Both the proximity and membership data are provided at 1Mb and 30kb resolutions.

### Obtaining 3D Genome Reconstructions

The focus of the present work is advancing methods for evaluating the accuracy of 3D genome reconstructions by taking advantage of newly devised assays: multiplex FISH and GAM. While the proposed methods are agnostic with respect to the technique used to effect the reconstruction, we illustrate ideas using the hybrid simulated annealing (HSA, [34]) algorithm. Beyond excellent performance in benchmarking studies, HSA has a number of compelling features. First, HSA is the only 3D genome reconstruction algorithm that can simultaneously integrate multiple data tracks. This capacity was developed in order to utilize the parallel contact matrices that are typically generated by Hi-C protocols corresponding to use of differing restriction enzyme digests. Here, however, we harness this facility by treating the abovementioned primary and replicate contact maps as tracks. Second, HSA adaptively estimates the power-law index whereby contacts are converted to distances, the importance of such adaptation having been previously emphasized [33]. Third, simulated annealing combined with Hamiltonian dynamics provides an effective optimization approach for exploring the high dimensional space representing the genomic loci’s 3D coordinates.

Like other 3D reconstruction algorithms [22, 29], HSA models (normalized) contact counts, *n*, via Poisson regression:

1 $$\begin{array}{@{}rcl@{}} n_{i_{k} j_{k}} &\sim & {Poi}\left(\mu_{i_{k} j_{k}}\right), \qquad k = 1, \ldots, K  \end{array} $$

2 $$\begin{array}{@{}rcl@{}} \ln\left(\mu_{i_{k} j_{k}}\right) &= & \beta_{k0} + \beta_{k1} \ln\left(d_{i_{k} j_{k}}\right)  \end{array} $$

3 $$\begin{array}{@{}rcl@{}} d_{i_{k} j_{k}} &= & || X_{i_{k}} - X_{j_{k}} ||_{2} \end{array} $$

where in (1) *k* indexes track, so that in some of our applications *K*=2 corresponding to primary and replicate data, and $n_{i_{k} j_{k}}$ is the count for genomic loci *i*_*k*_,*j*_*k*_. For notational simplicity we will impose that there are *n* common loci across tracks: *i*_*k*_=1,…,*n*;*j*_*k*_=1,…*n*;∀*k* although the HSA algorithm does not require this. The parameters *β*_*k*1_ correspond to (per-track) power-law indices relating expected counts (*μ*) to Euclidean distances (*d*). Provision exists for additional covariate terms (e.g. GC content, fragment length) to be included in (2) so as to accomplish in-line normalization. The $X_{i_{k}} = \left (x_{i_{k}},y_{i_{k}},z_{i_{k}}\right)$ and $X_{j_{k}} = \left (x_{j_{k}},y_{j_{k}},z_{j_{k}}\right)$ in (1) are the 3D coordinates for loci *i*_*k*_,*j*_*k*_ and are the (unknown) parameters constituting the reconstruction. These are subject to constraints owing to the local contiguity of chromatin. Zou et al. [34] capture these induced dependencies via a hidden Gaussian Markov chain. The full log-likelihood for *β*,*X* is then

4 $$ \begin{aligned} \ln(L\left(\beta,X | \mu, i_{k}, j_{k}\right) &\propto \sum_{k} \sum_{i_{k},j_{k}} \left[ - \exp \left(\ln\left(\mu_{i_{k} j_{k}}\right)\right. \right.\\ & \left. \left. + n_{i_{k} j_{k}} \left(\ln\left(\mu_{i_{k} j_{k}}\right)\right) \right) \right] \end{aligned}  $$

to which a penalty term controlling local smoothness is added. Note that (constrained) *X* enters (4) through *μ* and *d* from (2) and (1) respectively. The resulting penalized likelihood is optimized by iterating between generalized linear model (GLM, *cf* Poisson regression) fitting to obtain estimates $\hat \beta $ and simulated annealing to obtain estimates of the 3D coordinates $\hat X = \left (\hat x, \hat y, \hat z\right)$.

We note that the the GLM routine used (R function glm) does not require integral (count) data and so can accommodate normalized counts and GAM linkage disequilibrium scores. HSA subsumes several tuning parameters governing simulated annealing search. We have used default values throughout; however, the ability to compare accuracy of competing solutions as developed next provides a means for exploring differing tuning parameter settings.

### Accuracy Assessment

The two recent assays under consideration, multiplex FISH and GAM, provide distinct approaches to evaluating the accuracy of 3D genome reconstructions. Multiplex FISH imaging provides a gold standard from which the closeness of a 3D reconstruction can be measured, with inference making recourse to replicates. By deploying large numbers of thin nuclear cryosections reconstructions based on GAM assays admit accuracy assessment even in the absence of external gold standards. This results from the fact that the sectioning itself provides *geometric* information independent of derived distances and attendant reconstructions, as we detail below.

#### Multiplex FISH

We take the image-based 3D genomic coordinates furnished from multiplex FISH $\tilde X = \left (\tilde x, \tilde y, \tilde z\right)$ as the gold standard by which we evaluate our reconstruction solution $\hat X = \left (\hat x, \hat y, \hat z\right)$. In our approach three steps are necessary to effect such evaluation. First, we need to align (register) the reconstruction with the gold standard. This may involve preliminary coarsening of one or other coordinate set to yield comparable resolution. While subsequent Procrustes alignment (translation, rigid rotation and scaling [11]) is straightforward, issues surrounding loss (stress) symmetry are more involved and deferred to the Discussion. Second, we need a measure of agreement that quantifies how close the aligned reconstruction is to the gold standard, with (root) mean square deviation ((R)MSD), as deployed here, being the most widely used. Alternatives that operate on the underlying distance matrices [26], and so avoid alignment, are also addressed in the Discussion. Third, we need a scheme for arbitrating the adequacy of the measured agreement – it is for this typically challenging component that we provide methods customized to the multiplex FISH and GAM assays.

To simplify notation we let $\hat X_{i} = \left (\hat x_{i}, \hat y_{i}, \hat z_{i}\right)$ represent the scaled, aligned 3D genome reconstruction that we wish to compare with the gold standard $\tilde X_{i} = \left (\tilde x_{i}, \tilde y_{i}, \tilde z_{i}\right)$ at the set of *n* common genomic loci. Then the mean square deviation is given by

5 $$ \begin{aligned} \text{MSD}(\tilde X,\hat X) &= \frac{1}{n} \sum_{i=1}^{n} || \tilde X_{i} - \hat X_{i} ||^{2} = \frac{1}{n}\sum_{i=1}^{n} \left[(\tilde x_{i} - \hat x_{i})^{2}\right. \\& \quad\left.+ (\tilde y_{i} - \hat y_{i})^{2} + \left(\tilde z_{i} - \hat z_{i}\right)^{2}\right] \end{aligned}  $$

We obtain MSD values using the R package vegan [21] which also performs the preliminary Procrustes alignment of $\hat X$ to $\tilde X$. While extensive applications of (R)MSD in the world of 3D protein structure comparison have revealed concerns surrounding domination by largest deviations [14], our concern here is not with refinements to or selection of a particular agreement measure. Accordingly, the inferential scheme developed next can be applied with any measure substituted for the (R)MSD in (5).

3D *protein* structure comparisons have promulgated various prescriptions for evaluating agreement adequacy in terms of RMSD Ångström thresholds, although these have been called into question [18]. However, there is no basis for analogous thresholding of RMSD values in the unchartered context of 3D *genome* reconstruction comparisons, most of which do not provide configurations with an underlying physical distance. Instead, we seek appropriate (R)MSD referent distributions, developing differing approaches for multiplex FISH and GAM in accordance with data structure and availability.

For each chromosome we treat 3D coordinates provided by multiplex FISH as our gold standard $\tilde X$ and measure the MSD to our reconstruction $\hat X$ using (5). But, as noted, there are numerous multiplex FISH replicates for each chromosome, designated $\tilde X_{l}; l= 1, \ldots, L_{u}$, there being *L*_*u*_ replicates for chromosome *u*=1,…,*U*. Indeed, $\tilde X$ was obtained by locus-wise averaging over these replicates. To obtain a referent distribution for appraising $\text {MSD}(\tilde X,\hat X)$ we take advantage of these replicates and simply compute $\text {MSD}_{l} = \text {MSD}\left (\tilde X,\tilde X_{l}\right); l = 1, \ldots, L_{u}$. The resulting empirical distribution of MSD_*l*_ values captures experimental variation around the multiplex FISH gold standard. Interpretation of $\text {MSD}\left (\tilde X,\hat X\right)$ in the context of the MSD_*l*_ distribution is demonstrated in the Results.

A fine point is that, by construction, this distribution will exhibit reduced dispersion than its targeted population quantity (based on independently obtained $\left (\tilde X,\tilde X_{l}\right)$) owing to data re-use since $\tilde X_{l}$ contributes to $\tilde X$. While this concern could potentially be mitigated by employing a leave-one-out technique and utilizing a series of gold standards $\tilde X_{(l)}$ obtained by averaging over all replicates excluding $\tilde X_{l}$, the following considerations indicate this approach to be unnecessary: (i) the numbers of replicates involved is large (*L*_*u*_>110 ∀*u*) so that the contribution of individual chromosome replicates is modest; (ii) the imputation scheme used to handle missing coordinate data necessarily borrows strength across replicates, so even the leave-one-out scheme would not eliminate (complex but slight) dependencies of $\tilde X_{(l)}$ on $\tilde X_{l}$; (iii) the 3D reconstruction $\hat X$ is compared to $\tilde X$ so consistency requires comparing $\tilde X_{l}$ to $\tilde X$ and (iv) the impact of the reduced dispersion will be to make for more conservative inference.

We note that this approach to accuracy assessment relies on the availability of suitably large numbers of multiplex FISH image replicates. Absent such replication assessment of $\text {MSD}(\tilde X,\hat X)$ would require simulation, the inputs to which seem highly uncertain. Further, unlike the situation with GAM described next, the complex conformational dependencies present in 3D genome structures, preclude permutation or bootstrap resampling approaches.

#### GAM

We obtained 3D genome reconstructions for mESC chromosomes based on applying HSA to GAM linkage disequilibrium scores (at 1Mb resolution) as described above. As there are currently no public multiplex FISH imaging studies for mESCs, reconstruction accuracy assessment based on RMSD is precluded. However, information contained in locus membership in the collection of *planar* nuclear profiles (cryosections) can be used as follows.

The number of available nuclear profiles is 408, this number being sufficient to study chromatin architecture at 30kb at the sequencing depths deployed, as formally determined by power analysis [3]. The distribution of numbers of loci detected per chromosome per profile is clearly dependent on chromosome extent and positioning as we showcase in the Results. That the profiles are planar cross-sections is neither used in the determination of the normalized linkage disequilibrium score nor in the subsequent model [3] used to identify non-random loci interactions. However, it is central to our assessment of 3D reconstruction accuracy: by interrogating whether loci found in the same nuclear profile have coordinates that lie in a plane in the reconstruction we can gauge the extent to which the reconstruction preserves this physical property of the sectioning.

We make this proposal concrete by first using a measure of the degree to which a set of 3D points are planar, namely the proportion of variance explained by the sum of the first two principal components (corresponding to a planar projection), hereafter termed PC1+PC2 as given by the associated eigenvalues. We also consider the second principal component alone (termed PC2) in order to distinguish scenarios where the sum is dominated by the first principal component. Note that these measures are coordinate system-free, essential in view of the nature of 3D reconstruction solutions. Further, as some nuclear profiles may capture few or even zero loci, we restrict determination of PC1+PC2 and PC2 to those profiles with sufficient numbers of loci. By taking this to be approximately the upper quartile of the per chromosome per profile locus count we obtain 100 values of PC1+PC2 and PC2 for each reconstructed chromosome.

As was the case with multiplex FISH accuracy assessment we need a reference system for evaluating our PC scores. While there is an extensive body of work on random matrix theory (e.g. [1]) and attendant first eigenvalue distributions, the formulations thereof are inapplicable and results for second eigenvalues are lacking. To evaluate whether the PC scores measuring our 3D GAM-based reconstructions conform to planes corresponding to the nuclear profiles, we make recourse to permutation / sampling. Specifically, independently for each nuclear profile *j* (included in the top 100 locus counts) that contains say *n*_*j*_ loci, we sample *n*_*j*_ chromosomal loci, equivalent to permuting the loci indicator column vector of the segmentation matrix. We then obtain the 3D coordinates in the GAM-based reconstruction corresponding to these sampled loci and compute the PC scores for these. This amounts to assessing the planarity of *n*_*j*_ points in 3D that are randomly sampled from the reconstruction, with a view to contrasting such planarity summaries with corresponding summaries from the actual nuclear profiles. The contrasting is effected by performing the re-sampling a large number of times so as to provide a null distribution for the summaries. To confer robustness, we use ranking to summarize how the original PC scores compare to this null distribution which corresponds to computing an empiric *p*-value. A further level of summarization – over the 100 profiles – is required and, for robustness, we use the median.

## Results

### Multiplex FISH

**Fig. 1:**
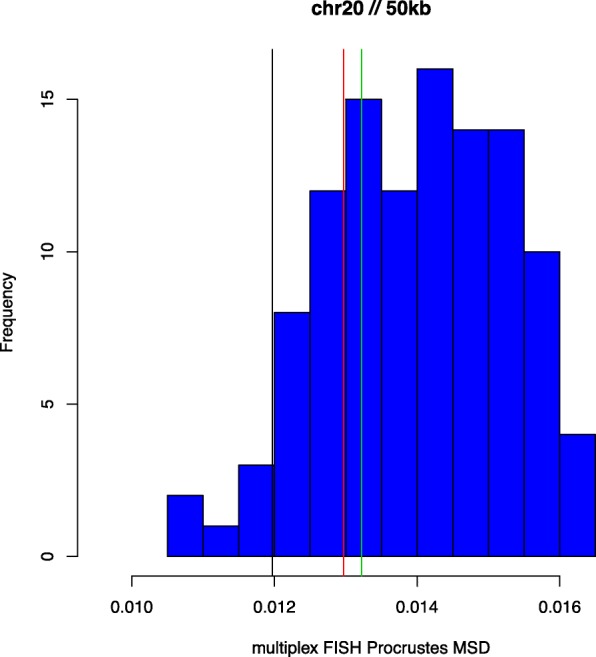
Multiplex FISH MSDs: Chromosome 20. The histogram (blue) depicts MSD_*l*_ values measuring agreement between multiplex FISH replicates $\tilde X_{l}$ and the mean configuration $\tilde X$. The respective vertical lines show MSD values from HSA reconstructions $\hat X$: primary data (red), replicate data (green), and combined data (black)

**Fig. 2:**
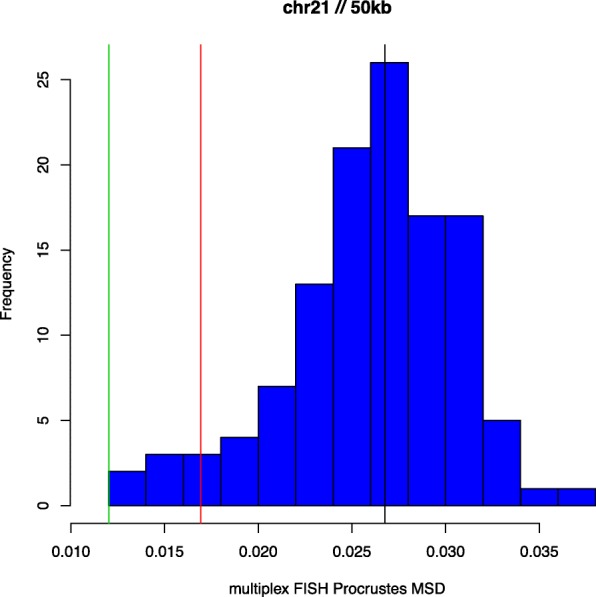
Multiplex FISH MSDs: Chromosome 21. As for Fig. 1

**Fig. 3:**
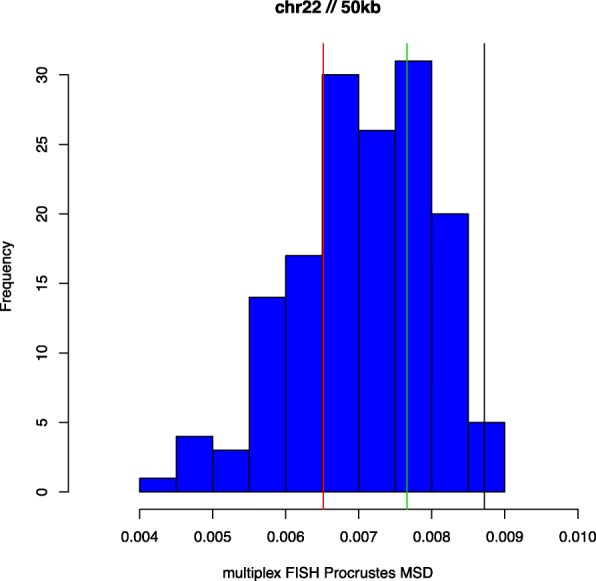
Multiplex FISH MSDs: Chromosome 22. As for Fig. 1

In Figs. 1, 2 and 3 we present histograms depicting the distribution of MSD_*l*_ values obtained using the multiplex FISH replicates as described above, for chromosomes 20, 21 and 22. Also shown are MSD values, derived using (5), from corresponding HSA 3D genome reconstructions. We use the capacity of HSA to perform multi-track fitting to obtain reconstructions for primary, replicate and combined data series. The results shown represent reconstructions from Hi-C data binned at 50kb resolution. While the multiplex FISH probe resolution of 100kb determines the effective overall resolution, the need to bin (generally higher-resolution) Hi-C data and the impact this can have on attendant reconstructions implies that accuracy assessments can be sensitive to resolution..

We first note that the three chromosomes studied differ appreciably in the variation of multiplex FISH replicates around their respective mean configurations, with chromosome 21 being the most and chromosome 22 being the least variable, as per the Procrustes MSD values. For chromosome 20 (Fig. 1) the respective HSA reconstructions conform not just with the multiplex FISH replicates but also with one another. For chromosome 21 (Fig. 2) we observe disparate behaviour between the HSA reconstructions, with that based on the replicate data series (green) being extreme relative to the multiplex FISH replicates, whereas the HSA reconstruction based on combined primary and replicate data series (black) conforms to the multiplex referent. For chromosome 22 (Fig. 3) we see the converse (to chromosome 21) behaviour, with the combined data being relatively extreme, while the primary and replicate series are more concordant with the multiplex FISH replicates. However, as indicated, the extent of variation for chromosome 22 is comparatively compressed. It is notable that for all chromosomes and all data series the HSA 3D reconstruction MSDs lie within the extent of multiplex FISH MSDs, indicating reasonableness of the 3D configurations derived from HSA. There is no guarantee that this obtains and, indeed, at other reconstruction resolutions HSA MSDs values outside the multuplex FISH range arise.

### GAM

Tables 1 and 2 present results from applying the program described above to all 19 mouse autosomes using GAM proximity (normalized linkage disequilibrium) measures at 1Mb and corresponding segmentation data that defines locus detection in each of the 408 nuclear profiles. We attempted to use the proximity matrices at 30kb resolution, however, there were numerous HSA convergence problems. Even at 1Mb resolution HSA reached the specified (default) maximal iteration count (100) for some of the smaller chromosomes (15 through 19).

**Table 1:** Rank of median (over the top 100 nuclear profiles) explained variances for PC1+PC2 and PC2 of the GAM-based HSA 3D reconstruction among an additional 1000 (within nuclear profile) permutations of loci [1 is least, 1001 is most explained variance]

	PC1 + PC2	PC2
Chrom 1	1	1000
Chrom 2	1001	1
Chrom 3	823	1001
Chrom 4	95	424
Chrom 5	991	1001
Chrom 6	1001	99
Chrom 7	1	1001
Chrom 8	1001	787
Chrom 9	1	220
Chrom 10	1001	1
Chrom 11	1	1001
Chrom 12	35	1001
Chrom 13	919	1
Chrom 14	11	395
Chrom 15	832	977
Chrom 16	859	1
Chrom 17	1001	116
Chrom 18	388	1
Chrom 19	717	95

**Table 2:** Median explained variance for first plus second principal components and second principal components of GAM-based HSA 3D reconstruction along with corresponding medians (and associated median absolute deviation (MAD)) based on 1000 (within-profile) loci permutations

	PC1 + PC2			PC2		
	Actual	Permuted	MAD	Actual	Permuted	MAD
Chrom 1	96.80	96.97	0.03	12.83	12.34	0.15
Chrom 2	89.11	88.30	0.15	33.23	34.26	0.26
Chrom 3	95.39	95.29	0.10	22.50	20.61	0.16
Chrom 4	93.85	93.97	0.10	23.60	23.65	0.25
Chrom 5	95.06	94.90	0.07	17.84	17.20	0.16
Chrom 6	94.70	94.52	0.06	18.25	17.66	0.24
Chrom 7	98.47	98.58	0.03	10.19	8.99	0.15
Chrom 8	81.08	79.25	0.18	30.79	30.48	0.40
Chrom 9	97.74	98.10	0.04	26.22	26.42	0.24
Chrom 10	88.96	87.82	0.16	34.84	36.46	0.21
Chrom 11	98.51	98.69	0.01	4.79	4.41	0.08
Chrom 12	94.06	94.26	0.12	26.50	25.03	0.21
Chrom 13	96.15	96.06	0.06	15.44	15.99	0.19
Chrom 14	99.23	99.26	0.02	3.47	3.48	0.05
Chrom 15	92.37	92.24	0.13	19.12	18.71	0.19
Chrom 16	98.07	98.04	0.03	32.92	33.27	0.03
Chrom 17	79.53	77.84	0.39	31.64	31.91	0.24
Chrom 18	93.60	93.63	0.13	38.07	38.82	0.18
Chrom 19	96.29	96.25	0.07	24.78	24.35	0.25

For each chromosome, and for each PC measure, the Tables 1 and 2 entries represent summaries over those 100 nuclear profiles that contain the largest number of genomic loci. These loci count upper quartiles range from *n*_1_=75 (chromosome 1) down to *n*_19_=27 (chromosome 19) and correlate (r = 0.97) with chromosome length. These counts represent the minimum numbers of loci per profile over the 100 nuclear profiles we utilize – they range up to maxima of *n*_1_=169 to *n*_19_=56. In Table 1, where we summarize via ranks, the ranking system is such that a result of 1001 indicates that the median (over the 100 selected nuclear profiles) eigenvalue of the principal component(s) fitted to the actual nuclear profiles exceeds all the corresponding median eigenvalues of the resampled profiles. Conversely, a result of 1 indicates that the actual nuclear profile median eigenvalue was smaller than the median for all of the resampled profiles.

Analogously, in Table 2 we present summaries of percent variance explained by the respective PCs. Thus, for example, for chromosome 2 the median percent variance – over the 100 selected nuclear profiles – for our measure of planarity, PC1+PC2, is 89.11%. This can be referenced to the value obtained under within-profile permutation of 88.30% with an associated MAD – over the 1000 permutations – of 0.15%. Corresponding values for PC2 alone are respectively 33.23%, 34.26% and 0.26. Values for PC1 can be obtained by subtraction.

Results for the 19 chromosomes reveal some interesting patterns and putative groupings. For several chromosomes (2, 5, 6, 8, 10, 17) attained PC1+PC2 median rankings are best – the reconstruction profiles have most, or near most, explained variance compared with resampling-based profiles – while some additional chromosomes (3, 13, 15, 16) are in the top 20%. These results all align with the percent variance explained summaries provided in Table 2 wherein the actual percent variance explained notably (as calibrated by the MAD) exceeds that of the permuted value. The interpretation of such a finding is that the chromosome’s 3D reconstruction is in excellent agreement with the planarity represented by the (top 100) nuclear profiles. What is striking, however, is the set of chromosomes for which the planar explained variance is minimal (chromosomes 1, 7, 9, 11) or near minimal (chromosomes 12, 14) – in most of these instances the second principal component alone is dominant. Interpretation here is uncertain, with the results potentially being driven by the nature of the resampling scheme. In particular, in view of local chromatin contiguity, it may be desirable to constrain the within-profile loci considered and resampled to those that are separate with respect to genomic distance.

## Discussion

In this paper we have exploited two recent biotechnologies, multiplex FISH with TAD-based probes and genome architecture mapping, in order to assess the accuracy of 3D genome reconstructions. As indicated in the Introduction, such reconstructions can confer added value with respect to downstream biological insight, but clearly any putative insights are conditional on the quality of the reconstruction. Gauging quality has proven exceedingly difficult accentuating the need for methods such as those proposed here. Both these methods, and the new assays themselves, can be refined and deployed in additional ways.

As well as being used for accuracy assessment, multiplex FISH can also be used to improve the actual 3D reconstructions via better calibration of the power-law transfer function (e.g. [29]) for converting Hi-C contacts to distances, which is a prelude to many 3D reconstruction approaches. This potential can be appreciated by contrast with the only existing tool for performing calibration, FisHiCal [27]. There, 4 FISH distances, from probes straddling over 1Mb, were used to estimate 3 transfer function parameters, this being inherently unstable. As multiplex FISH uses ∼30 structurally relevant probes targeting the centers of 100-kb TADs yielding a far richer ($\sim \binom {30}{2} = 435$) and higher resolution distance set, simply following the FisHiCal prescription will yield substantial improvements in calibration accuracy. Beyond this, the extent of paired distance : contact data will allow formulation of more sophisticated transfer functions, enabling known power-law deficiencies [17, 19] to be overcome. Additionally, the existence of observed 3D scaffolds [30] from the multiplex FISH probes may better facilitate incorporation of constraints into the constrained optimization approaches for obtaining 3D reconstructions [2, 9].

Our use of multiplex FISH to gauge the accuracy of Hi-C based 3D genome reconstructions is predicated on its constituting a suitable gold standard. One limitation to use of multiplex FISH is resolution disparity with Hi-C data being available at much higher resolutions. Another issue is that Hi-C counts can be driven by factors beyond spatial proximity, such as access to the nuclear compartment outside the chromosome territory core [31] as well as the potential that Hi-C and FISH are accessing differing cell subsets [7] and may be differentially influenced by loops [10, 12], complicates this perspective. However, given that FISH and Hi-C proximities are largely concordant, we believe that the use of *multiplex* FISH, with the associated substantive increase in probe numbers and resolution, mitigates these concerns. Moreover, we have accommodated variation in multiplex FISH imaging in adopting it as a referent. Additionally, we have relied upon (R)MSD as a measure of configuration closeness. Other metrics are available, with distanceError being frequently used in the context of 3D genome reconstruction [25, 26, 29] with related single distance [22] and correlation [16, 33] measures also being deployed. These approaches are putatively more robust to outliers than (R)MSD, yet can require standarization to achieve scale invariance. For (R)MSD scale difference are handled via estimation of a corresponding (linear) scaling parameter as part of Procrustes alignment. It should be noted that by designating a gold standard (target) configuration this scaling is asymmetric with respect to the multiplex FISH and Hi-C reconstruction and forcing symmetry (equal configuration dispersion) distorts the interpretation of the (R)MSD statistic.

GAM ostensibly offers several advantages over Hi-C [3]. Whether these extend to 3D genome reconstruction accuracy awaits more extensive uptake since, at present and like multiplex FISH, a primary limitation is the extent of available data. The approaches to accuracy assessment developed here, based on the planarity of numerous cryosections for GAM and replicates for multiplex FISH, will gain broader applicability as these novel assays are used in additional settings.

## Conclusion

We have developed methods for assessing the accuracy of 3D genome reconstructions that exploit features of recently advanced multiplex FISH and genome architecture mapping assays. These approaches can help overcome the absence of gold standards for making such assessments which are important in view of the considerable uncertainties surrounding 3D genome reconstruction. R code implementing multiplex FISH- and GAM-based accuracy assessment is available on github: https://github.com/marksegal/reconstruct-accuracy.

## Abbreviations

3D: three dimensional
FISH: Fluorescence in situ hybridization
GAM: Genome architecture mapping
GEO: Gene expression omnibus
GLM: Generalized linear model
HSA: Hybrid simulated annealing
mESC: Mouse embryonic stem cells
MDS: Multi-dimensional scaling
(R)MSD: (Root) mean squared deviation
PC: Principal component
TAD: Topologically associated domain

## References

[1] Anderson GW, Guionnet A, Zeitouni O (2010). An Introduction to Random Matrices.

[2] Ay F, Bunnik EM, Varoquaux N, Bol SM, Prudhomme J, Vert JP, Noble WS, Le Roch KG (2014). Three-dimensional modeling of the P. falciparum genome during the erythrocytic cycle reveals a strong connection between genome architecture and gene expression. Genome Res.

[3] Beagrie RA, Scialdone A, Schueler M, Kraemer DC, Chotalia M, Xie SQ, Barbieri M, de Santiago I, Lavitas LM, Branco MR, Fraser J, Dostie J, Game L, Dillon N, Edwards PA, Nicodemi M, Pombo A (2017). Complex multi-enhancer contacts captured by genome architecture mapping. Nature.

[4] Capurso D, Segal MR (2014). Distance-based assessment of the localization of functional annotations in 3D genome reconstructions. BMC Genomics.

[5] Capurso D, Bengtsson H, Segal MR (2016). Discovering hotspots in functional genomic data superposed on 3D chromatin configuration reconstructions. Nucleic Acids Res.

[6] Cusanovich DA, Daza R, Adey A (2015). Multiplex single-cell profiling of chromatin accessibility by combinatorial cellular indexing. Science.

[7] Dekker J (2016). Mapping the 3D genome: Aiming for consilience. Nat Rev Mol Cell Biol.

[8] Dixon JR, Selvaraj S, Yue F, Kim A, Li Y, Shen Y, Hu M, Liu JS, Ren B (2012). Topological domains in mammalian genomes identified by analysis of chromatin interactions. Nature.

[9] Duan Z, Andronescu M, Schutz K, McIlwain S, Kim YJ, Lee C, Shendure J, Fields S, Blau CA, Noble WS (2010). A three-dimensional model of the yeast genome. Nature.

[10] Fudenberg G, Imakaev M (2017). FISH-ing for captured contacts: towards reconciling FISH and 3C. Nat Methods.

[11] Hastie TJ, Tibshirani RJ, Friedman JH (2009). The Elements of Statistical Learning.

[12] Imakaev MV, Fudenberg G, Mirny LA (2015). Modeling chromosomes: Beyond pretty pictures. FEBS Lett.

[13] Kalhor R, Tjong H, Jayathilaka N, Alber F, Chen L (2011). Genome architectures revealed by tethered chromosome conformation capture and population-based modeling. Nat Biotech.

[14] Kufareva I, Abagyan R (2012). Methods of protein structure comparison. Methods Mol Biol.

[15] Lee CS, Wang RW, Chang HH, Capurso D, Segal MR, Haber JE (2016). Chromosome position determines the success of double-strand break repair. Proc Natl Acad Sci.

[16] Lesne A, Riposo J, Roger P, Cournac A, Mozziconacci J (2014). 3D genome reconstruction from chromosomal contacts. Nat Meth.

[17] Lieberman-Aiden E, van Berkum NL, Williams L, Imakaev M, Ragoczy T, Telling A, Amit I, Lajoie BR, Sabo PJ, Dorschner MO, Sandstrom R, Bernstein B, Bender MA, Groudine M, Gnirke A, Stamatoyannopoulos J, Mirny LA, Lander ES, Dekker J (2009). Comprehensive mapping of long-range contacts reveals folding principles of the human genome. Science.

[18] Maiorov VN, Crippen GM (1994). Significance of root-mean-square deviation in comparing three-dimensional structures of globular proteins. J Molec Biol.

[19] Mateos-Langerak J, Bohn M, de Leeuw W, Giromus O, Manders EMM, Verschure PJ, Indemans MHG, Gierman HJ, Heermann DW, van Driel R, Goetze S (2009). Spatially confined folding of chromatin in the interphase nucleus. Proc Natl Acad Sci.

[20] Nagano T, Lubling Y, Stevens TJ, Schoenfelder S, Yaffe E, Dean W, Laue ED, Tanay A, Fraser P (2013). Single-cell Hi-C reveals cell-to-cell variability in chromosome structure. Nature.

[21] Oksanen J, Blanchet FG, Friendly M, Kindt R, Legendre P, McGlinn D, Minchin PR, O’Hara RB, Simpson GL, Solymos P, Stevens MHH, Szoecs E, Wagner H. vegan: Community Ecology Package. R package version 2.4-1. 2016. http://CRAN.R-project.org/package=vegan.

[22] Park J, Lin S (2017). A random effect model for reconstruction of spatial chromatin structure. Biometrics.

[23] Ramani V, Deng X, Gunderson KL, Steemers FJ, Disteche CM, Noble WS, Duan Z, Shendure J (2017). Massively multiplex single-cell Hi-C. Nat Methods.

[24] Rao SS, Huntley MH, Durand NC, Stamenova EK, Bochkov ID, Robinson JT, Sanborn AL, Machol I, Omer AD, Lander ES, Aiden EL (2014). A 3D map of the human genome at kilobase resolution reveals principles of chromatin looping. Cell.

[25] Rousseau M, Fraser J, Ferraiuolo MA, Dostie J, Blanchette M (2011). Three-dimensional modeling of chromatin structure from interaction frequency data using Markov chain Monte Carlo sampling. BMC Bioinformatics.

[26] Segal MR, Xiong H, Capurso D, Vazquez M, Arsuaga J (2014). Reproducibility of 3D chromatin configuration reconstructions. Biostatistics.

[27] Shavit Y, Hamey FK, Lio P (2014). FisHiCal: an R package for iterative FISH-based calibration of Hi-C data. Bioinformatics.

[28] Tjong H, Gong K, Chen L, Alber F (2012). Physical tethering and volume exclusion determine higher-order genome organization in budding yeast. Genome Res.

[29] Varoquaux N, Ay F, Noble WS, Vert JP (2014). A statistical approach for inferring the 3D structure of the genome. Bioinformatics.

[30] Wang S, Su J-H, Beliveau BJ, Bintu B, Moffitt JR, Wu C-T, Zhuang X (2016). Spatial organization of chromatin domains and compartments in single chromosomes. Science.

[31] Williamson I, Berlivet S, Eskeland R, Boyle S, Illingworth RS, Paquette D, Dostie J, Bickmore WA (2014). Spatial genome organization: contrasting views from chromosome conformation capture and fluorescence in situ hybridization. Genes Dev.

[32] Witten DM, Noble WS (2012). On the assessment of statistical significance of three-dimensional colocalization of sets of genomic elements. Nucleic Acids Res.

[33] Zhang Z, Li G, Toh K-C, Sung W-K (2013). 3D chromosome modeling with semi-definite programming and Hi-C data. J Comp Biol.

[34] Zou C, Zhang Y, Ouyang Z (2016). HSA: integrating multi-track Hi-C data for genome-scale reconstruction of 3D chromatin structure. Genome Biol.

